# The Evolution of Telehealth From Pre-COVID-19 Pandemic Through A Hybrid Virtual Care Delivery Model: A Pediatric Hospital's Journey

**DOI:** 10.5195/ijt.2021.6432

**Published:** 2021-12-16

**Authors:** Evelyn Abrahante Terrell, Saima Aftab, Anne Babitz, Lauren Butler, Nicole Gondar Hernandez, Bianca Hornik, Keysla Lee, Jennifer Perez, Elizabeth Sotolongo, Jessica Thomas

**Affiliations:** 1 Nicklaus Children's Pediatric Virtual Care, Telehealth Center, Nicklaus Children's Hospital, Miami, Florida, USA; 2 Department of Rehabilitation Services, Nicklaus Children's Hospital, Miami, Florida, USA

**Keywords:** COVID-19, Early intervention, Pediatrics, Rehabilitation services, Telehealth

## Abstract

The COVID-19 pandemic transformed care delivery and influenced telehealth adoption by rehabilitation professionals and their patients. The purpose of this paper is to describe a pediatric health system's telehealth services pre-pandemic and how those services were scaled during the pandemic. A secondary aim is to provide a roadmap for the operational delivery of telehealth and rehabilitation services, including transition to a hybrid care delivery model. Findings suggested that telehealth can be rapidly scaled to address patient healthcare needs for an early intervention population during a pandemic. Telehealth use during the pandemic helped ensure continuity of care and likely reduced the risk of exposure to patients and staff to the virus. Benefits included enhanced access to care, and savings in time and money for families. Interestingly, as the pandemic declined, the use of telehealth services declined due to patient preference, with many families opting to request a return to in-person care.

Telehealth has proven to be a valuable tool in managing the pediatric population, including high-risk children with medical complexity, by avoiding unnecessary trips to the hospital and preventing potential exposure to communicable diseases (Notario et al., 2019; Onofri et al., 2021; Ross et al., 2020). The American Occupational Therapy Association (AOTA) defines telehealth as “the application of evaluative, consultative, preventative, and therapeutic services delivered through information and communication technology” (AOTA, 2018). Studies suggest that telehealth services can be as effective as in-person care (Bican et al., 2021; Krasovsky et al., 2021) and are a cost-effective delivery model (Longacre et al., 2020; Nissen et al., 2018). Telehealth has been proposed as a way to increase accessibility and enhance continuity of care for vulnerable populations with disabilities (Hailey et al., 2011). This led to virtual rehabilitation program development in an attempt to provide equitable access to therapy for individuals who live in remote geographic locations and who are physically or economically disadvantaged (Theodoros et al., 2008). Crow et al. (2009) found that cognitive behavioral therapy delivered via telehealth had similar efficacy to in-person treatment, while demonstrating lower costs. Cost savings in that study were largely related to a reduction in travel costs. Furthermore, services delivered via telehealth were positively perceived by both patients and clinicians. In a systematic review by Kairy et al. (2009), patients and therapists reported positive perceived benefits, convenience, and usefulness of the telehealth program.

Telehealth is also an effective model for identifying at-risk children for early intervention (EI) services to ensure early detection of developmental problems and to enhance access to care for optimal outcomes (ASHA, 2021; Cason et al., 2012). EI services are offered to eligible infants and toddlers, ages birth to 36 months with disabilities, developmental delays, and at-risk conditions in the areas of physical, cognitive, communication, social-emotional, and/or adaptive domains (Early steps, 2021). EI services are provided to the family and child by implementing developmentally appropriate learning opportunities during everyday activities and routines. EI best practices includes parent coaching, and research suggests that treatments can be integrated into the child's natural environment (Kronberg et al., 2021; Wallisch et al., 2019). Research has shown that EI, using evidence-based practice, is effective in improving long-term outcomes for children with Autism Spectrum Disorder (ASD) and that telehealth is a promising delivery model for this population (Baharav & Reiser, 2010; Boisvert, et al., 2010; Boisvert, et al., 2012). A single-subject, multiple-baseline design study by Vismara et al. (2012) examined the feasibility and acceptance of telehealth by nine families that each had a child with ASD. Results demonstrated significant improvement in parent fidelity over time during treatment (X^2^=342.58, df=1, P<.001, d=4.62) (Vismara et al., 2012). Similarly, the use of telehealth to deliver EI speech language pathology (SLP) services has been found to result in significant increases in behaviors that promote social and communication interactions, with participants showing improvements in all domains measured during home-based sessions facilitated by the parents (Baharav & Reiser, 2010).

Rehabilitation services provided to the EI population include occupational therapy (OT), physical therapy (PT), and SLP services. When conducting an evaluation for rehabilitative services, clinicians must consider the patient's health care needs, preferences, and access to technology. Outcomes must be measurable (AOTA, 2018; Tohidast et al., 2020). Several outcomes are achievable though telehealth and include improved participation in activities of daily living, health and wellness, role competence, well-being, and quality of life (AOTA, 2018; Bican et al., 2021).

Prior to the COVID-19 pandemic, patients and families were hesitant to consider telehealth as an option due to limited experience, knowledge, and awareness of its reliability and effectiveness (Peretti et al., 2017; Wood et al., 2021). Providers were also reluctant to embrace telehealth as a service delivery model that might provide quality services and best practices to their patients (Peretti et al., 2017; Wood et al., 2021). Additionally, health systems struggled to implement telehealth programs and with sustainability of services due to lack of reimbursement and limited viable business models (Tomines, 2019).

The purpose of this paper is to describe a pediatric health system's EI services pre-pandemic and how those services were scaled during the pandemic. This paper will provide a roadmap for the operational delivery of telehealth and rehabilitation services, and for transition to a hybrid model.

## TELEHEALTH CONSULTATIVE PROGRAM PRE COVID-19 PANDEMIC

In 2014, the Department of Rehabilitation Services for a pediatric healthcare system launched a telehealth program for children who were part of one of 15 statewide EI programs. This came out of a needs assessment that identified an underserved rural county that included 13 cities and towns. This community faced many challenges that included long travel distances to services, internet challenges, and lack of access to pediatric specialists. When the program launched in the rural county there were 71 cases approved for EI services and only four rehabilitation professionals who were contracted to provide in-person services to children.

Between January 2014 and August 2017, the telehealth team serviced over 105 patients via a telehealth consultative model. A total of 76% of telehealth consults were speech and language pathology services, 13% were physical therapy services, and 11% were occupational therapy services. The telehealth program provided access to rehabilitation professionals including OTs, PTs, and SLPs for telehealth consultations to the Infant Toddler Developmental Specialist (ITDS) in the child's home or daycare setting. An ITDS is a non-licensed provider of EI services that focuses on designing learning environments to promote infant/toddler development *(Florida's Early Steps System.* 2020). The ITDS, in consultation with other EI providers on the child and family's team, assists the family in understanding the special needs of their child to enhance the child's development. Due to the limited availability of specialized professionals in the rural county, the ITDS lacked resources to support children in achieving optimal functional outcomes within their natural environments. In alignment with the EI model and as suggested by Akhbari Zeigler and Hadder-Algra (2020), our focus was to ensure that services remained within the natural environment for optimal carryover.

The American Academy of Pediatrics (AAP) recommended developmental surveillance and administration of standardized screening tests at nine months, eighteen months, and thirty months and at the four and five-year well visit (Lipkin & Macias, 2020). An identified problem should result in evaluation, diagnosis, counseling, treatment and/or EI (Lipkin & Macias, 2020). The identified targets for this consultative model were children who qualified for the state EI program and resided in an underserved area. Through coaching delivered via the telehealth platform, we were able to offer caregivers effective therapies for eligible children and families who may not have otherwise received services. We understood that to maximize outcome measures and programmatic success, active family integration of therapy activities was essential (Wallisch et al., 2019).

Our pediatric healthcare system embraced the opportunity to remove barriers to care by providing telehealth consultative services to providers and families who resided in identified rural communities. Part of this journey was to collaborate with our community partners to address challenges and barriers, develop workflows, and create solutions that were meaningful to the ITDS, therapists, families, and patients.

Using the telehealth platform, our pediatric rehabilitative therapists guided the ITDS in performing techniques that assisted the child in gaining functional outcomes in their natural environment. This telehealth consultative service delivery model offered equitable access to rehabilitative services in the areas of consultation and training for the home-based provider and family, as outlined in the child's Individualized Family Service Plan (IFSP) (IDEA, 2017). Through collaboration with the EI system, we facilitated the coaching required for effective carryover within the natural environment. The coaching strategy used for the telehealth model allowed for collaboration between the coach, in this case the rehabilitation therapist who was remote, and the on-site parent and/or ITDS. This partnership allowed for a true family-centered approach that supported each child's unique individual needs throughout the therapeutic process, within the child's natural setting (Landinguin, 2020).

Over the course of the program's span, the majority of completed telehealth consults consisted of first-time sessions, which subsequently became recurring patient care via this service delivery model. It is important to note that a recipient of this service delivery model had the option of having services provided either within their home, or within their child's childcare center. Of the two options, the home was the most requested at 78%. The outcomes of the telehealth services were not specifically evaluated or measured.

**Figure 1:**
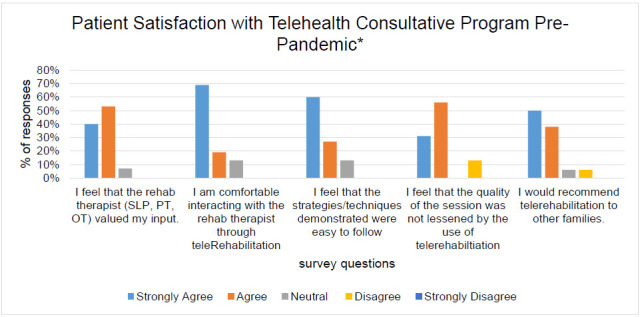
Patient Satisfaction with Telehealth Consultative Program Pre-Pandemic

*Note.* *data collected from 2015 through 2017

Through the implementation of the telehealth consultative model, cost savings were estimated at $96 per consult totaling $10,080 for all provided consults. This was a result of travel reduction and reduced time away from work for the caregiver. In 2014, a survey was provided to all caregivers of telehealth services. The survey consisted of questions specific to overall experience, technology experience, and cohesiveness of therapeutic session. Patient satisfaction was determined based on behavioral questions presented via a Likert scale 1-5 (one (1) Strongly Agree and five (5) Strongly Disagree). Patient satisfaction ratings are shown in Figure 1.

At the end of 2017, the local regional EI agency underwent provisional changes which impacted the telehealth program between the health system and the community partners (ITDS). At that time, the health system lost the necessary funding to continue services with the local regional EI agency. As a result of these changes, the EI agency transitioned all services from a virtual consultation service delivery model back to direct patient care rendered solely by the ITDS provider. This was a return to the process followed prior to the health system's launching of telehealth services in 2014.

## COVID-19 RESPONSE

In response to the COVID-19 pandemic, the public was encouraged to seek medical care remotely for non-emergent medical concerns. As a result, healthcare systems and providers were proactive in transitioning many of their services to telehealth with the goal of promoting social distancing, minimizing the risk of exposure, and ensuring continuity of patient care. The rapid scaling of telehealth services became necessary to meet patient needs and minimize exposure by keeping patients at home when possible.

To better serve the community and continue to provide timely and high-quality pediatric care, providers expanded their reach and telehealth technology solutions. This allowed health systems to conserve valuable personal protective equipment (PPE) resources early in the pandemic, helping to decrease the burden of disease transmission and reduce community spread of COVID-19. Telehealth services were especially valuable for the population of complex and chronically ill children, many of whom are severely immunocompromised (Harper, 2006; Ignatowicz et al., 2019).

The COVID-19 Public Health Emergency (PHE) temporary waivers and flexibilities eliminated many of the regulatory requirements and barriers that had prevented telehealth expansion for decades, thereby accelerating adoption by all users, including providers and patients. Key drivers and benefits that facilitated expansion included the reduction in exposure to COVID-19 by keeping patients and providers safe at home, enhanced access, and continuity of care (Wood et al., 2021). COVID-19 PHE waivers and flexibilities included the relaxation of legislation and improved reimbursement, which allowed for rapid expansion of telehealth services and expedited utilization by patients and providers (AHA, 2020).

As a result, we were able to initiate telehealth services for EI agencies within our region. However, the service delivery model changed from consultation to direct patient care. Prior to the pandemic the services were delivered from the therapist to the ITDS, whereby the therapist guided the ITDS in therapeutic strategies to meet patient goals. During the pandemic, this model transitioned to direct virtual patient care, whereby the therapist worked with the patient and family independent of the ITDS. To organize the effort to expand existing virtual care services and prepare for the increase in virtual care volumes, our health system responded to the pandemic in 3 phases: Phase 1: Initial crisis response, Phase 2: Virtual care expansion, and Phase 3: Recovery and beyond." The full operational plan can be viewed in Table 1 below.

**Table 1:** Operational Plan

Activity	Deliverable description
**Phase 1 (March 2020-April 2020)**	
**Telehealth platform enhancements**	Completed telehealth platform enhancements, connectivity requirements and testing. Included user account creation and testing.
**Telehealth / technology solution installation / delivery /support**	Deployed devices to providers to enable telehealth visits while working from home and office settings. Created user accounts and passwords. Conducted testing on connectivity and provided training to patients and families on the application. Trained personnel at distant sites on use of equipment by their patients. Provided on-going training and support to all users.
**Community partner engagement**	Began engaging community partner sites and developed workflows/processes to launch telehealth services.
**Phase 2 (April 2020-June 2020)**	
**Telehealth platform roadmap**	Telehealth platform review was completed and process improvement plan was initiated following Lean methodologies. Developed platform roadmap based on recommendations from users and subject matter experts for future enhancements to usage, connectivity, and effectiveness.
**Telecommunications and information services expansion**	Expanded network, bandwidth, and systems that allowed patients and providers to most easily connect via video. Increased the number of patients that could be cared for virtually. Information services included internet connectivity services for healthcare providers and their patients.
**Develop data collection reports and program dashboard**	Developed program specific dashboard to measure, track, and trend data specific to pre-determined program metrics and key performance indicators.
**Phase 3 (June 2020 to present)**	
**Implement new programs and services in the home**	Conducted test and real-time virtual visits for underserved and vulnerable populations.
**Community partner expansion**	Developed and expanded existing workflows and processes to launch telehealth services with additional community partner sites.

### PHASE 1 – INITIAL CRISIS RESPONSE

During the initial mandated governmental closures, the health system received communication from various EI agencies that it was imperative to continue services and maintain continuity of care during the state of emergency. As a result, the agency was expanding coverage of therapy services and EI services provided through telehealth. This initiated the development and use of the standardized hospital Situation – Background - Assessment – Recommendation (SBAR) communication tool with the partnership of a multidisciplinary team inclusive of rehabilitation leaders, chief physicians, managed care leaders, health information management leaders, administrative leaders, and legal team. The SBAR allowed concise communication to address the request to quickly launch telehealth services for children who were previously receiving EI services in-person in our outpatient hospital setting across two counties. Once the SBAR was approved by the hospital's incident command team the Rehabilitation Services Department quickly deployed a systematic operation plan to launch telehealth services to those who qualified for EI services.

The Telehealth Center had been operational since 2013, with at least one provider from each participating physician subspecialty area granted telehealth privileges. During the initial COVID-19 crisis response phase, the Telehealth Department focused all efforts on expanding virtual care services to specialties and providers. The team worked closely with all specialty areas to identify workflow needs, provide clinician training, and clarify reimbursement and documentation needs.

Goals of the initial crisis response phase were to expand the audio-visual (AV) and other capabilities of the telehealth platform, and to expand ongoing relationships with regional EI programs. This allowed rehabilitation clinicians the opportunity to provide the community with access to OT, PT, and SLP services for those quarantined or confined due to the COVID-19 pandemic. This was particularly important for the medically complex, geographically, and socioeconomically isolated pediatric population. A secondary goal was to ensure continuity of care and reduce the risk of exposure for both healthy and high-risk patients and their families by keeping them out of the hospital, as well as protecting staff and healthcare providers. To accomplish these goals, we developed two primary areas of focus: technology platform solutions and virtual care services.

#### TECHNOLOGY PLATFORM SOLUTIONS FOR TELEHEALTH SERVICES

Initially, prior to clinician training, technology needs were assessed to ensure providers had the minimum necessary equipment to conduct virtual visits. Equipment requirements included a laptop or PC, Google Chrome browser, webcam with microphone, and speakers as well as a private space to conduct virtual visits. Based on requirements, a telehealth technology readiness survey was completed across all outpatient centers.

A rehabilitation task force was then created, trained, and on-boarded to the platform. The telehealth team worked closely with the Lean/ Process Improvement Department to identify and implement the initial platform enhancements. The health system had a synchronous proprietary telehealth platform that was developed in 2013. Changes or updates to the application design were made through collaboration with the Information Technology (IT)/Web Development Department. The enhancements made in this phase focused mainly on improving the patient experience. Enhancements included removing the requirement for patients to validate their email prior to connecting with a provider, limiting patient clicks by minimizing account creation screens, and adding pop-ups which displayed connectivity tips. Additional enhancements included working with the platform's cloud provider to support the increase in call volumes and allow the application to host more sessions simultaneously. The changes made during this initial phase resulted in a better experience for patients and their families.

#### CLINICAL LAUNCH FOR TELEHEALTH SERVICES

The rehabilitation task force targeted the following key areas to launch telehealth services: billing, documentation and coding, scheduling, patient identification for telehealth, and provider training. Given differences in billing, coding, and documentation between telehealth service delivery models and in-person care, the rehabilitation team worked with the IT, Revenue Integrity, and Managed Care departments to ensure that documentation and billing within the electronic medical record (EMR) were updated to allow for added billing code modifiers and appropriate clinical documentation. This required a new billing section to indicate that services were provided using a telehealth model, and to append billing code modifiers. Clinicians providing telehealth services were trained on how to use the new telehealth billing section, and the charge reconciliation practices to support proper billing.

Newly designated scheduling slots and scheduling rules were created to ensure that telehealth appointments would be scheduled only with telehealth-trained clinicians. Appointment durations for telehealth visits were also set to allow for adequate time for clinicians and patients to sign-in to their electronic devices. This allowed time to troubleshoot any technical difficulties that could arise without creating scheduling delays that would impact adjacent appointments on the clinician's schedule.

To identify which patients were appropriate candidates to receive rehabilitation services via the telehealth model, the rehabilitation team created a list of all EI identified patients across all outpatient centers. This roster was used to contact families, inform them of available telehealth services, and confirm their interest in receiving rehabilitation services via this model. The list included services (OT, PT, SLP) authorized, frequency recommendations, and caregiver contact information for ease of registration and scheduling.

The rehabilitation task force assumed ownership over training their providers as well as tracking training completion, readiness, and competencies, to ensure successful utilization of the telehealth platform. The rehabilitation task force met with each clinician to provide guidance on room set-up, how to access the hospital network remotely, how to download the appropriate telehealth software on their computer, and how to obtain a camera and microphone for their computer if they did not already have one. Provider access to the telehealth platform was tested and verified prior to the delivery of care using a 'simulated training session.' Providers were also given a coaching strategy guide, a telehealth training manual, and a caregiver instruction manual to guide their learning. Workflows were developed and provided to assist clinicians in determining a patient's eligibility to receive telehealth services, and offer guidance on how to schedule telehealth visits. Workflows can be found in Appendix A and Appendix B. During the initial roll-out of telehealth services, training was provided to 15 providers and then expanded to include 120 providers.

### PHASE 2 – VIRTUAL CARE EXPANSION

Virtual care expansion followed the initial crisis response phase. During the expansion period, workflows were enhanced from a process improvement perspective, and additional resources were made available to both patients and providers.

#### TECHNOLOGY PLATFORM ENHANCEMENTS

As more patients and providers began utilizing the telehealth platform, we began collecting feedback from users. Subsequently, a telehealth strategy team made up of clinical and non-clinical staff from different specialty areas was formed. The team met monthly to discuss and brainstorm platform enhancements, best practices, and the overall virtual care experience. Based upon the readily available data from surveys and feedback from patients, providers and their teams, a telehealth roadmap was developed to display the requested enhancements and upgrades. During this phase a call center was created to assist all users, including providers and families, with any telehealth technology needs and COVID-19 related health information. The call center used a web-based application that allowed multiple agents to connect with several users simultaneously. The web-based application generated reports; these allowed for the tracking of incoming call response times, call center volumes, and abandoned call rates. This information determined appropriate call center staffing needs.

#### CLINICAL EXPANSION OF TELEHEALTH SERVICES

During the pandemic, rehabilitation providers continued to scale telehealth services. Patients received scheduled virtual visits based on recommendations from the child's plan of care and initial evaluation. The highest volume of services delivered to children continued to be within the birth to 36-month age group (i.e., those served under the EI model).

To benefit our families, it was important that providers incorporated the standards of practice for each discipline while using telehealth as a platform. Telehealth providers have a duty to practice in a manner that is both consistent with their scope of practice and congruent with the prevailing standards of practice for in-person services (Richmond et al., 2017). Focus was placed on processes to initiate therapy and intervention models and evaluation techniques, to discern barriers and successes for the implementation of clinical components within the telehealth model (Wallisch et al., 2019).

#### INITIATION OF THERAPY (PROCESS FOR FIRST SESSION WITH EACH PATIENT)

Prior to the initiation of treatment, providers completed thorough chart reviews to ensure understanding of patients' current level of function, frequency, and duration of plan of care, recommendations, and goals. Registration personnel called the family 15 minutes prior to their scheduled session to assist with any sign-on issues and to check-in the patient to enable documentation and billing at the end of the session. At the start of the first session, providers introduced themselves, explained the expectation of virtual care, and ensured the family's comfort during their first experience with virtual care. Providers focused on building rapport with the patient and family and reviewed the family's goals for treatment.

#### INTERVENTION AND EVALUATION

To optimize progress when working with children who are birth to 36 months of age, it is important to provide education, early intervention, and support to families. Previous literature has reported that parents can be effective in improving their child's developmental skills. According to Rakap and Rakap (2014), when parents used naturalistic strategies, the child's language skills were shown to improve. This further supported the need to provide services via telehealth during the pandemic which tied in with the EI motto: “services are provided to the family and child where they live, learn and play, to enable the family to implement developmentally appropriate learning opportunities during everyday activities and routines” *(Florida's Early Steps System.* 2020). Various evidence-based practices exist to inform rehabilitation professionals how to provide direct intervention to children and how to coach parents (Akhbari Ziegler & Hadders-Algra, 2020; Comeau, 2020; Kemp & Turnbull, 2014; Snodgrass et al., 2017).

As telehealth services continued on a recurring basis, providers and families discussed the materials and equipment required for future sessions. This allowed families to prepare using readily available household items and toys in order to facilitate efficient and effective sessions. Providers coached caregivers on how to set-up the natural environment so that they addressed targeted goals within their daily routines. Feedback to both improve interaction and support the acquisition of skills were also provided to the caregiver. Throughout the intervention journey, it was equally important for the provider to understand the learning styles of both the patient and the caregiver. Modeling of intervention techniques and strategies was used to strengthen the relationship between the caregiver and providers. During sessions, providers discussed goals and progress along with strategies and exercises to work on between sessions. As patients met goals, providers planned for discharge with families, shared ideas on how to continue incorporating learned strategies during daily routines, and reviewed expected milestones and progress.

Providers completed virtual evaluations and re-evaluations in alignment with the plan of care timelines utilizing standardized measures, checklists, parent questionnaires, and observations. In order to complete the virtual evaluation in the home environment, providers guided caregivers on gathering appropriate materials needed for the evaluation. In-person evaluations and re-evaluations were offered for medically complex patients or those deemed necessary by the provider.

#### SUCCESSES AND BARRIERS

Through the virtual care implementation journey, rehabilitation services providers and families encountered both successes and barriers. Feedback from providers suggested that several caregiver factors contributed to success. These included: consistent attendance, high engagement, acceptance of feedback, and carryover of strategies within the natural environment. Utilization of routine activities, highly engaged patients, and providers who were adaptable to different caregiver learning styles were also identified as positive factors for therapeutic success. Barriers to success were also reported and included connectivity issues that made it difficult to maintain participation in front of the camera, difficulty with camera placement, caregiver fatigue, failure of the caregiver to prepare activities for the upcoming sessions, difficulty engaging the child, and difficulty obtaining signed plans of care from physicians to continue services.

### PHASE 3 – RECOVERY & BEYOND

As the health system re-opened and elective procedures and in-person visits became more readily available, we entered the recovery and beyond phase. This phase consisted of implementing a hybrid approach to care delivery which included a combination of in-person and virtual visits. Recognizing that telehealth is not a “one size fits all” approach, families were given the choice of which model they wished to participate in; their preference was aligned to our existing episodic care models. Each family that was receiving telehealth services was contacted and informed that they were able to either return to in-person care, continue with virtual visits, or choose a hybrid model that included both in-person and virtual visits. If insurance limitations prohibited the family from receiving telehealth services, they were offered either self-pay or redirected to in-person care.

The telehealth team and collaborating departments monitored shifting regulations related to patient privacy, data security, and insurance reimbursement to ensure that the platform adhered to new requirements. An insurance matrix was developed by the Managed Care Department to provide all clinical departments with updates regarding insurance reimbursement for telehealth.

As existing telehealth services continued to evolve, telehealth platform enhancements, Lean process improvement methodologies and tools were embedded. As a result, the teams were able to measure, analyze, improve upon, and sustain workflows and processes for optimal user experience and to create more value for the customer (AHRQ, 2017). Lean methodologies were utilized to measure key performance indicators and the effectiveness of process improvement steps that were implemented (Shortell et al., 2021). Our methods focused on the following: (1) Journey mapping through the customer experience and virtual care workflow, (2) Creating data visualization tools that allowed all data to be viewed in one location via a dashboard, and (3) Use of lean tools. The Lean tools utilized included: the use of the swim lane process to map out existing workflows in greater detail, the creation of fishbone diagrams to identify potential root causes of problem areas, and pareto analysis which helped us identify a course of action by quantifying the benefits of addressing our problem areas (Shortell et al, 2021).

#### TECHNOLOGY PLATFORM SUSTAINABILITY

During this phase, required enhancements were identified and a telehealth roadmap was developed. The roadmap included future upgrades such as EMR integration, rapid-join encounter capabilities, and new features and functionalities resulting in an enhanced user (i.e., patient and provider) experience. As the roadmap comes to completion, the teams will continue to evaluate additional improvements that will contribute to creating a high-quality experience for our patients. Currently, the virtual care platform and the EMR do not communicate and require providers to login to each separately. Rapid-join and integration enhancements will allow the provider to join the virtual session and connect to the patient from one link within the medical record. Patients will also be able to join the encounter from a link that is sent to them via text message prior to their appointment, and they will no longer be required to create an account.

#### TRANSITIONING TO A HYBRID MODEL FOR TELEHEALTH SERVICES

During this transition, the rehabilitation service line continued to assess the needs of patients based on the changing environment. Scheduling teams notified families of the center's reopening and provided the option of continuing telehealth or returning to in-person care. Families considered safety concerns, the health status of their child (i.e., if medically fragile/immuno-compromised), their schedules, convenience, and the potential for increased progress resulting from in-person care in the clinic setting. Families then chose to either stay with the telehealth model or return to in-person care at the center. Some families chose a hybrid model. Hybrid models varied and included combinations of care such as: one weekly appointment delivered via telehealth and remaining appointments in-person; alternating in- person and telehealth weekly; and weekly telehealth sessions with re-evaluations in-person. An in-person evaluation was often recommended and preferred by providers (Hsu et al., 2021). Providers felt that an in-person evaluation allowed for a more thorough assessment of patient deficits due to poor adaptability of some standardized pediatric assessment tools to the home environment. As the pandemic evolved, providers continued discussions with their families to assess the appropriate model for therapy.

Operationally, the rehabilitation service line encountered the need to create appointments labeled as tele-treatment versus in-person treatment in order to notify the provider and recipients of the service delivery model. Providers were assigned two, one-hour telehealth blocks per week and these blocks were mixed in with in-person care appointments. One-hour blocks were standard to account for connectivity issues that could arise.

As restrictions were lifted and community COVID-19 cases declined, more families chose to return to in-person care with fewer patients opting for telehealth. This required more options for in-person appointment slots and reduced the need for telehealth blocks. Ultimately, the designated telehealth treatment blocks were removed from providers' schedules. Telehealth appointments were scheduled within the regular treatment slots and labeled as “tele-treatment.”

It is the authors' opinion that this preference for in-person service may have stemmed from families' pre-pandemic familiarity and comfort with in-person services. This trend suggests the need for further evaluation of the telehealth delivery model to better understand factors that impact adoptability in the pediatric population.

### OUTCOMES

#### KEY PERFORMANCE INDICATORS

Several metrics and key performance indicators were tracked to evaluate the program's success and determine if the program's goals and objectives were achieved. A review involved secondary analysis of existing clinical databases. Services were monitored on an on-going basis by a comprehensive data collection system and dashboard that tracked volumes and visits, diagnoses, demographics, and patient satisfaction comments about telehealth services. This data collection system included the EMR, patient satisfaction surveys, and a hospital developed travel tool. All data were collected from April 2020 through September 2021.

An application was submitted to the Research Institute and the Human Protections Administrator for Non-Human Subjects Research Determination. The committee determined this to be a quality improvement project that did not meet the definition of research (AHRQ, 2017). Thus, IRB approval was not necessary.

## RESULTS

### VOLUMES AND VISITS

**Figure 2:**
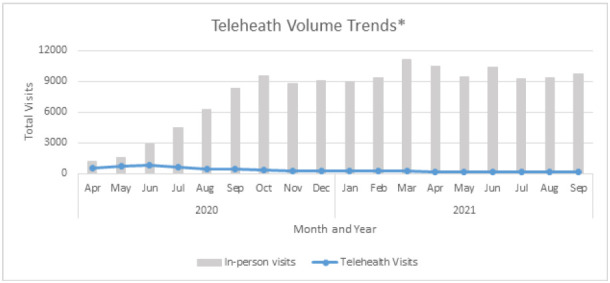
Telehealth Volume Trends

*Note.* *data collected from April, 2020 through September 2021

In response to the COVID-19 pandemic, we were successful at rapidly scaling telehealth services. We launched virtual services with 565 rehabilitation virtual visits provided in the month of April 2020, representing 49% of all visits provided by rehabilitation professionals for that month. From April 2020 through September 2021, rehabilitation service professionals provided 5,677 virtual visits, with 4,343 virtual visits provided in 2020 and 1,334 provided in 2021 (Figure 2).

**Figure 3:**
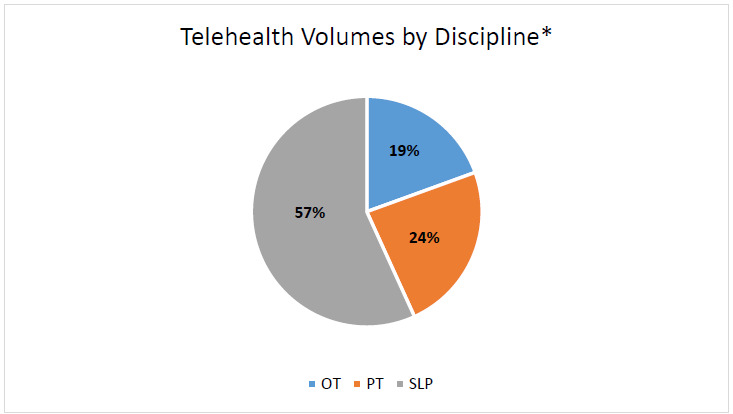
Telehealth Volumes by Discipline

*Note.* *data collected from April, 2020 through September 2021

Total visits from OT accounted for 19%, PT accounted for 24% and SLP accounted for 57% of total virtual visits provided. Total rehabilitation virtual visits by discipline are shown in Figure 3.

**Figure 4:**
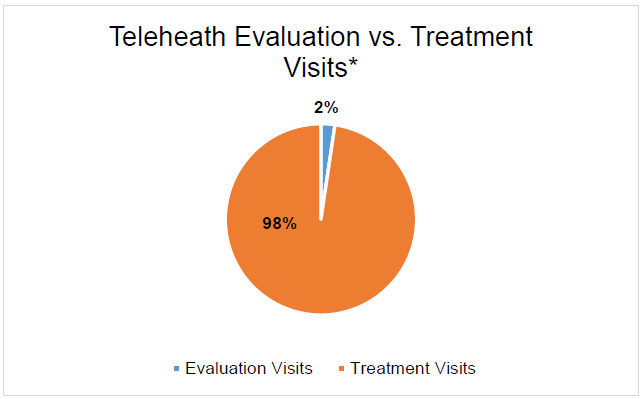
Telehealth Evaluation vs Treatment Visits

*Note.* *data collected from April 2020 through September 2021

When analyzing evaluation and treatment visits, we found higher numbers of treatment visits compared to evaluation visits across all disciplines (Figure 4.).

### DIAGNOSES AND DEMOGRAPHICS

A total of 273 patients were seen for PT, OT, and SLP from April 2020 through September 2021. We saw more male patients compared to female patients (n= 161 and n = 112 respectively). The mean age of patients treated was 3.1 + 3.2 years. The most common diagnosis treated was developmental disorder of speech and language, unspecified (n= 45). Patient demographics and diagnoses treated are summarized in Table 2.

**Table 2:** Age and Gender by Diagnosis and Specialty

Diagnosis (ICD-10* Code)	Total n	Male n (%)	Female n (%)	Mean Age
**Overall**	273	161 (59%)	112 (41%)	3.1 ± 3.2
Developmental disorder of speech and language, unspecified (F80.9)	45	30 (67%)	15 (33%)	3.1 ± 2.7
Unspecified lack of expected normal physiological development in childhood (R62.50)	31	17 (55%)	14 (45%)	2.3 ± 2.4
Specific developmental disorder of motor function (F82)	19	12 (63%)	7 (37%)	2.2 ± 1.8
**Physical Therapy**	79	43 (54%)	36 (46%)	2.4 ± 2.8
Specific developmental disorder of motor function (F82)	13	7 (54%)	6 (46%)	1.8 ± 0.9
Unspecified lack of expected normal physiological development in childhood (R62.50)	12	8 (67%)	4 (33%)	3.2 ± 3.5
Delayed milestone in childhood (R62.0)	10	4 (40%)	6 (60%)	1.5
**Occupational Therapy**	73	41 (56%)	32 (44%)	3.3 + 3
Unspecified lack of expected normal physiological development in childhood (R62.50)	12	5 (42%)	7 (58%)	1.5
Delayed milestone in childhood (R62.0)	9	4 (44%)	5 (56%)	1.5
Specific developmental disorder of motor function (F82)	8	6 (75%)	2 (25%)	6.7 ± 3.1
Autistic disorder (F84)	8	6 (75%)	2 (25%)	3.3 ± 2.5
**Speech therapy**	121	77 (64%)	44 (36%)	3.5 ± 3.4
Developmental disorder of speech and language, unspecified (F80.9)	45	30 (67%)	15 (33%)	3.2 ± 3.2
Unspecified lack of expected normal physiological development in childhood (R62.50)	16	7 (44%)	9 (56%)	1.7 + 0.8
Feeding difficulties (R63.3)	15	9 (60%)	6 (40%)	1.7 + 0.8

* *Note*. ICD-10 = International Statistical Classification of Diseases and Related Health Problems, 10 Revision

### PATIENT SATISFACTION

The pediatric health system had a hospital-wide patient satisfaction survey administered by Press-Ganey which captures the “voice of the customer” and measures the patient experience (Jehle, et al., 2020). Patients were chosen to complete the survey via random selection, following the Hospital Consumer Assessment of Healthcare Providers (HCAHPS) survey method, which does not require informed consent (Davidson et al., 2017; Lappé, et al., 2020).

Telehealth patient satisfaction surveys were launched for physician practices in partnership with Press-Ganey to align with the hospital-wide survey process. Telehealth surveys received through April 2021 for physician practices showed 88% overall satisfaction. A telehealth specific survey was not available for rehabilitation services; however, there was already an established Press-Ganey patient satisfaction survey in place that was administered for all rehabilitation service patient types (in-person and virtual). A key word search of survey results related to rehabilitation visits that took place from the time that virtual services were launched in April, through September 2021, revealed that there were no negative comments that mentioned “virtual,” “online,” or “tele,” visits, and that the majority of the comments were positive. Sample comments can be viewed in Table 3. The complete survey can be found in Appendix C.

**Table 3:** Rehabilitation Services Telehealth Satisfaction Survey Results

Patient Satisfaction Telehealth Sample Comments
“I truly appreciate how supportive all of the PT's have been during COVID. I am so thankful for the ability to have tele appointments!!”
“Huge credit to *Clinician Name* for being the most incredible PT! For telerehab. Going above and beyond for us.”
“Online therapy is working out great for *Patient Name*.”
“Always good! *Clinician Name* works very well with my son with his online therapy sessions.”
“A lot of our appointments have been virtual due to COVID.”

### TRAVEL SAVINGS

The Health System developed a “Distance and Time Travelled Tool” leveraging a Directions Application Programming Interface (API), which is a pre-packaged machine learning algorithm to calculate total miles, total time saved, and estimated fuel cost in order to capture overall cost-savings for our patients. Our results demonstrate total cost savings for our patients and families of $44,562, including 269,257 total miles, and 6,410 hours total saved.

## DISCUSSION

The COVID-19 pandemic has amplified existing healthcare disparities and pediatric populations are particularly at risk due to the narrow developmental window for impact of EI and rehabilitation services (Akhbari Ziegler & Hadders-Algra, 2020). In the United States, the ever-ballooning healthcare expenditures and shrinking healthcare work force have created additional challenges. Widespread and ongoing use of telehealth in the pediatric rehabilitation space offers exciting potential to improve access and outcomes and lower the cost of care, while allowing flexibility to healthcare workers and reducing their risk of burnout. Evolving changes and regulatory shifts from the COVID-19 pandemic have impacted the telehealth landscape and future virtual care delivery. Telehealth advocates representing the interests of rehabilitation professionals are collaborating to ensure that the Centers for Medicare and Medicaid (CMS) extend waivers beyond the duration of the COVID-19 PHE. The goal is to ensure that the substantial advancements toward telehealth adoption are not lost, and to facilitate access and continuity of care for patients.

Some important considerations for policymakers include the elimination of restrictions in provider type and practitioner eligibility for delivery of telehealth services beyond temporary waivers. This will enhance provider autonomy and ensure access to non-physician providers, including rehabilitation professionals. Additionally, policymakers must remove originating site restrictions, to allow the patient's home to serve as the originating site for telehealth. Lastly, coverage, reimbursement and equitable payment for telehealth services by federal, state and commercial insurers of rehabilitation services is a top priority to ensure health equity and access to critical services (AOTA, 2021; APTA, 2021; ASHA, 2021; ATA, 2021).

In order to advance permanent telehealth reform, the sustainability of telehealth services, through the creation of hybrid (in-person and virtual) models of care beyond the pandemic are recommended. Further expansion of telehealth and the development of more robust hybrid care models may provide more equitable access to care and reduce health care disparities (Wood et al., 2021). Additionally, innovative models of telehealth delivery, such as professional-to-professional consultation, should be considered for underserved populations with limited access to specialists. The feasibility and benefits of telehealth including improved access to services, improved access to specific providers and specialists, and reduction in delay of care have been previously reported (Cason, 2014; Olson et al., 2018; Utidjian & Abramson, 2016).

Telehealth has been shown to provide positive clinical results with lower cost, improved convenience, and reduced health risk exposure (Olson et al., 2018; Suso-Martí et al., 2021). Virtual care can improve patient satisfaction and address health equity challenges, paramount to ensuring optimal outcomes and access to quality services for all people (Krasovsky et al., 2021; Little et al., 2021).

Our findings showed that telehealth visits for rehabilitation accounted for almost half of all visits upon program launch in April 2020, however, by June 2020 that number had decreased to less than 30%. As opportunities for in-person rehabilitation visits increased, the demand for telehealth visits decreased exponentially, with less than 5% of rehabilitation visits provided via telehealth by year's end. Future works should explore opportunities to improve telehealth adoptability and sustainability in pediatric populations.

Generally, rehabilitation providers and families expressed positive feedback regarding both the telehealth only model and hybrid model of care, both of which continue. Providers gained valuable insight into the natural environment of families; this was helpful to achieving carry-over and generalization of therapeutic strategies. One limitation in our data is that we were unable to administer a telehealth patient satisfaction survey specific to rehabilitation. Given the need for rapid scaling of telehealth services due to the COVID-19 pandemic, there was not enough time to implement a unique survey for telehealth. The data presented were extracted from a general patient satisfaction survey. Future studies should specifically evaluate patient satisfaction with telehealth rehabilitation services.

It is currently unknown which populations benefit the most from the telehealth delivery model; this is particularly true for the pediatric population. A meta-analysis by Suso-Marti et al. (2021) found that telehealth demonstrates similar clinical results compared to in-person services for patients with neurological and musculoskeletal conditions, however, the same was not shown for patients with cardiac and respiratory conditions. Our experience showed that the top three diagnoses seen for telehealth rehabilitation services were related to developmental delay and disorders within the birth to 3-years age group. However, this distribution could be attributed to the fact that the majority of the patients that were seen for telehealth were from the EI program. The highest representation across the rehabilitation disciplines consisted of developmental disorders of language, resulting in the highest utilization of services within speech-language pathology. Higher utilization of telehealth in the SLP discipline may be related to referral patterns for therapeutic services from the EI program. Future work should aim to determine the effectiveness of rehabilitation telehealth services across different pediatric diagnoses and age ranges.

Travel related cost savings demonstrated within our telehealth model included total miles, total time saved, and estimated fuel cost. Factors related to cost savings not accounted for may include reduced time off work for travel to appointments, missed work or school days, reduction in childcare costs associated with appointments, public transportation, parking, and practitioner-related costs. Additionally, future studies should aim to determine the effect of the telehealth delivery model on factors such as duration and frequency of care, improved quality of life, and functional outcomes.

Future research should aim to utilize a framework to systematically evaluate components of telehealth service delivery and build evidence to demonstrate its effectiveness as we shift to a value-based care model (Cason, 2015). The PACE Framework (i.e., population and health outcomes, access for all patients, cost and cost effectiveness, experiences of patients and occupational therapy practitioners) has previously been suggested as a useful model to collect and analyze data related to telehealth outcomes (Chuo et al., 2020; Little et al., 2021). Factors such as reduction in healthcare disparities, parental stress, diversity, equity and inclusion, availability of specialists, reduction in patient wait times, digital health literacy, and patient satisfaction should all be considered in future frameworks and investigations.

## CONCLUSION

This report describes the rapid expansion of telehealth EI services during the COVID-19 pandemic in a pediatric health system. We present the steps taken and the guidelines developed from the initial crisis response through the development of a hybrid care delivery model. Our experience highlights the feasibility of rapidly scaling existing telehealth models to meet crisis needs for rehabilitation services in the EI population. It also highlights the challenges and opportunities of transitioning to a hybrid care model.
